# Detection of breast cancer lesions using APT weighted MRI: a systematic review

**DOI:** 10.1186/s12967-025-06153-7

**Published:** 2025-01-31

**Authors:** Ryan C. Lee, Montek Singh Boparai, Tim Q. Duong

**Affiliations:** Department of Radiology, Montefiore Medical Center and Albert Einstein College of Medicine, Bronx, NY USA

**Keywords:** Breast cancer, Amide Proton transfer, Chemical exchange saturation transfer, MRI

## Abstract

Amide proton transfer (APT) is a novel magnetic resonance imaging (MRI) technique that has shown promising ability to study cancers. This paper systematically reviewed the literature on the use of APT MRI in the prognosis of breast cancer. A literature search was conducted on Pubmed and Embase and a total of 14 articles comprising 775 patients were included in the review. APT MRI had the ability to distinguish between benign and malignant lesions with an AUC as high as 0.959. There is a positive correlation between APT signal intensity and tumor grade/stage as well as Ki-67, whereas no correlation was found with ER/PR/Her-2 receptor status. There was a greater decrease in APT signal intensity after neoadjuvant chemotherapy (NAC) in responders compared to non-responders, suggesting that APT MRI may serve as a valuable supplemental tool in the early identification of chemotherapy response. APT has the potential to complement with other imaging methods in the diagnosis, prognosis, treatment monitoring, and management of breast cancer. Additional studies and standardization of APT acquisition methods are needed.

**Supplementary Information:**

The online version contains supplementary material available at 10.1186/s12967-025-06153-7.

## Background

Breast cancer is the leading cancer diagnosis and second leading cause of cancer deaths in women in the United States [1]. It is a highly heterogeneous disease whose diagnosis, treatment, and prognosis are heavily influenced by various factors including receptor status (such as estrogen (ER), progesterone (PR) receptor), pathologic grade, and even Ki-67 level [2, 3].

Magnetic resonance imaging (MRI) is the standard imaging method for diagnosis and monitoring of disease progression and treatment outcomes because it is non-invasive, offers excellent soft tissue contrast, and excellent spatiotemporal resolution [4–6]. Common breast MRI methods include Dynamic Contrast-Enhanced MRI (DCE-MRI), Diffusion-Weighted Imaging (DWI), T2-Weighted Imaging (T2WI), and less commonly used methods such as MR elastography and MR spectroscopy. However, contrast agents are frequently required in the prior approaches which may present renal complications and contrast deposition in tissue [7, 8].

**Fig. 1:**
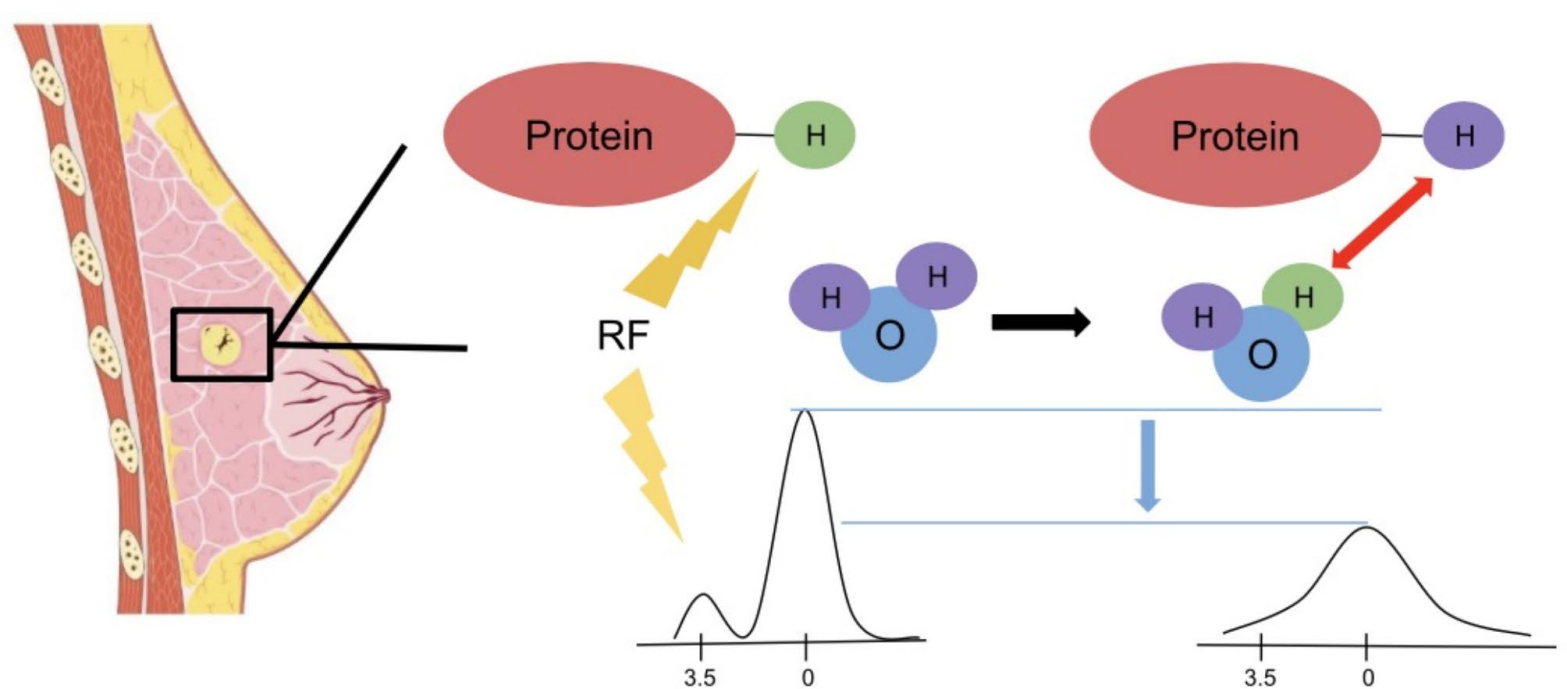
APT weighted imaging principle. A radiofrequency is applied at the specific frequency of amide protons 3.5ppm downfield the water signal. The exchange of amide protons with free water protons causes a change in the water signal. Asymmetry analysis around ± 3.5ppm is analyzed as the APT weighted signal and reported as MTRasym

Chemical exchange saturation transfer (CEST) has been introduced as a novel contrast method which measures the exchange of protons between amide, amine, hydroxyl, and water groups [9]. Of note, it does not require external contrast agents, which may expand its usefulness among patients with renal complications. While there are various approaches to CEST that measure different functional groups, a specific subset of CEST measures the amide proton transfer (APT) at 3.5ppm away from the resonance frequency of water and has been widely investigated in various cancers [10–12]. Amide proton transfer weighted imaging is sensitive to endogenous mobile proteins and peptides in tissue as opposed to the hydrogen atoms in free water which is measured in many other MR approaches. The principles and theories of CEST and APT weighted imaging have been reviewed in numerous articles [13–18]. In brief, the APT signal is observed as a decrease in the water signal caused by the exchange of water protons with tagged amide protons in mobile proteins. This provides indirect information about specific proteins through the water signal typically analyzed in imaging. The tagging is achieved by applying selective radiofrequency (RF) waves at the specific frequency of amide protons, approximately + 3.5 ppm downfield of the water signal. The data is analyzed using a Z-spectrum, which shows how the water signal changes with and without RF saturation. However, effects such as direct water saturation (DS) and magnetization transfer contrast (MTC) can interfere with the measurements. These effects are separated using asymmetry analysis, which calculates the difference in the Z-spectrum at + 3.5 ppm (amide protons) and − 3.5 ppm (other effects), reported as MTRasym. A schematic of APT-weighted imaging is provided in Fig. 1.

Since the 2000s, researchers have investigated the potential for APT imaging in cancer diagnosis. In preclinical tumor models, increases in cytosolic protein concentrations have been found and correlated to increases in APT signal intensity [19, 20]. A further study has found that approximately 66% of an APT signal change in tumors can be attributed to protein concentration differences as malignant tumors typically have increased free protein due to angiogenesis and hypercellularity [21]. This theory can be applied clinically as APT has been shown to be able to distinguish malignant from benign lesions, predict certain histologic grades, and correlate positively with response to neoadjuvant chemotherapy (NAC) response. APT weighted imaging applications have predominantly been investigated in brain tumors [22]. It has also been investigated in other cancers including salivary gland cancers, head and neck cancers, and even secondary metastases [23–25]. Its use in clinical practice is steadily increasing, which has led to the development of consensus recommendations for standardization across different centers and MRI vendors for the study of brain tumors [22].

In recent years, a few studies have investigated the use of APT MRI in breast cancer. Like studies in other cancers, they have also centered around distinguishing malignant from benign lesions, evaluating tumor characteristics, and predicting NAC response. Comprehensive review consolidating the body of research on APT MRI application to breast cancer is lacking. We thus conducted this systematic review to assess the diagnostic and prognostic use of APT in breast cancer and its future clinical applications.

## Methods

### Eligibility criteria

**Fig. 2:**
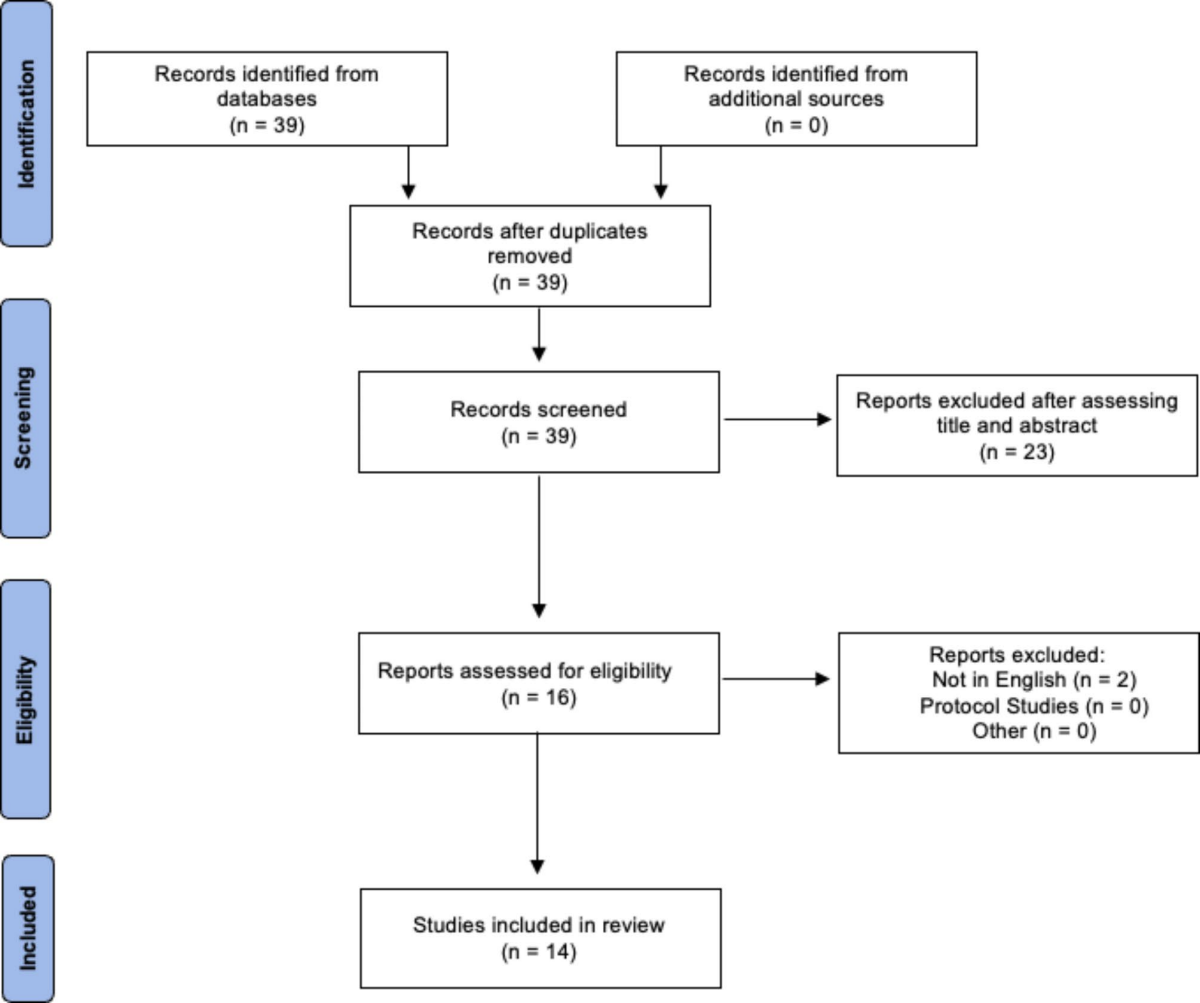
PRISMA diagram demonstrating the study inclusion criteria

Using Preferred Reporting Items for Systematic Reviews and Meta-analyses (PRISMA), we conducted a systematic review of studies that analyzed the use of APT in identifying breast cancer lesions (Fig. 2). A search was performed on PubMed and Embase databases and articles up to July 8 2024 were analyzed. The search parameters can be found in the supplemental information. Cohort studies and case series were included in the analyses. The following study types were excluded: (1) case reports, review papers, and conference abstracts; (2) papers not written in English; (3) protocol papers, letters to the editor, preprint papers, and healthcare provider surveys without data; and (4) papers that did not use APT as a data metric.

The title and abstract of papers after the initial search were assessed by two independent reviewers, M.B and R.L, and only studies approved by both reviewers were included. Disputes regarding the inclusion of a paper were decided by a third reviewer, T.D.

### Data collection and analysis

Study characteristics including study design, origin, sample size, and lesion characteristics as well as outcomes such as APT signal intensity and histopathological data were collected manually by two independent reviewers. The Newcastle-Ottawa Scale (NOS) was used to assess the quality of each study and the risk of bias. The results can be found in the supplemental information.

## Results

### Study characteristics

Thirty-nine unique papers were identified through PubMed and Embase searches. After review of abstracts, 25 studies were excluded for the following reasons: article was not written in English (2), article was not cohort or case series study (5), study did not use APT as a metric in the study of human breast cancer (18) (Fig. 2). The study characteristics and main findings of the 14 included papers are shown in Table 1. In summary, there were 10 prospective cohort studies, 3 retrospective cohort studies, and 1 case series. In total, 775 patients underwent an APT MRI scan, of which there were 568 malignant lesions and 214 benign lesions. All malignant lesions were verified by histology. Additional information regarding the average size of the lesions and histopathological data including ER status, PR status, HER2 status, Ki67%, and histological grade can be found in the Supplemental Data (Supplemental Table 1).

**Table 1:** Paper characteristics

Author Country, Year	Design	Patients (*N*)	Benign/Malignant Lesions (*N*)	Main Findings
Zhuang L China, 2023 [26]	P	40	14/28	- Malignant lesions had a significantly higher APT than benign lesions - MTRasym (APT) had similar efficiency to TIC (DCE)
Kamitani T Japan, 2023 [27]	R	66	0/66	- Triple negative tumors had higher signal intensity - Positive correlation between Ki67 and signal intensity
Liu Z China, 2023 [28]	P	84	16/68	- Malignant lesions had a significantly higher APT than benign lesions - Stage 1(TNM grading) showed lower APTw signals stage 2 or 3 - Positive correlation between Ki67 and signal intensity
Meng N-1 China, 2021 [29]	R	133	59/76	- Benign lesions had a significantly higher APT than malignant lesions - Positive correlation between histologic grade and signal intensity - DKI was superior to APT
Zhang N-1 China, 2022 [30]	P	56	15/41	- Malignant lesions had a significantly higher APT than benign lesions - Negative correlation between ER and signal intensity - APT had similar efficacy to DCE and was superior to DWI
Zhang S USA, 2021 [31]	P	51	0/51	- APT decreased in both responders and nonresponders of NAC, but it could not differentiate between the two groups
Li Y China, 2024 [32]	P	78	43/37	- Benign lesions had a significantly higher APT than malignant lesions - Positive correlation between histologic grade and signal intensity - APT had similar efficacy to DCE
Yu T China, 2024 [33]	P	52	8/44	- Malignant lesions had a significantly higher APT than benign lesions - APT had similar efficacy to DKI and IVIM
Zhang N-2 China, 2024 [34]	P	50	0/50	- APT signal change in NAC responders was greater than nonresponders
Meng N-2 China, 2020 [35]	R	121	59/62	- Benign lesions had a significantly higher APT than malignant lesions - Positive correlation between histologic grade and signal intensity - APT had similar efficacy to IVIM
Dula AN USA, 2013 [36]	CS	3	0/3	- Case series of 3 NAC patients (2 responders/1 nonresponder)
Loi L Germany, 2020 [37]	P	10	0/10	- Malignant lesions had significantly higher APT than healthy tissue (no benign lesions in this study) - Moderate positive correlation between Ki67 and signal intensity
Krikken E-1 Netherlands, 2018 [38]	P	9	0/10	- Significant signal change in responders before and after NAC - Could not distinguish responder from nonresponder change in NAC
Krikken E-2 Netherlands, 2019 [39]	P	22	0/22	- Paper looked at the pH relationship to APT. Found a decrease in pH led to increase in APT

P = prospective cohort; R = retrospective cohort; CS = case series

### Distinguishing benign vs. malignant lesions

Eight studies reported the APT signal values for benign and malignant lesions (Table 2). Four studies reported a significantly higher APT signal in malignant lesions, 3 studies reported significantly higher APT signal in the benign lesions, and 1 study only reported APT signal for only malignant lesions. It should be noted that 2 of the studies that reported a higher APT signal in benign lesions (Meng 1 [29] and Meng 2 [35]) may have overlapping patient cohorts. Seven studies reported an APT signal cut-off value for differentiating benign and malignant lesions. Of the papers that reported a higher APT signal for malignant lesions, Zhang et al. [30] reported the highest AUC of 0.959. Of the papers that reported a higher APT signal for benign lesions, Li et al. [32] reported the highest AUC of 0.816.

**Table 2:** APT values of benign vs. malignant lesions, sensitivity, specificity, AUC

Paper	APT Signal					
	Benign Lesions	Malignant Lesions	Cut-Off	Sens (%)	Spec (%)	AUC
Zhuang L	2.01 ± 0.51	3.18 ± 1.07*	> 2.35	85.71	92.86	0.915
Liu Z	0.54 ± 1.13	1.55 ± 1.24*	NR	77.9	62.5	0.716
Meng N-1	5.53 ± 1.09	4.13 ± 1.34*	< 5.260	84.2	67.8	0.796
Zhang N-1	1.50 ± 0.54	3.21 ± 1.04*	> 2.30	100	90.2	0.959
Li Y	2.68 ± 1.19	1.19 ± 0.82*	< 2.42	86.5	67.6	0.816
Yu T	1.22 ± 2.10	3.67 ± 1.59*	> 1.523	95.5	62.5	0.810
Meng N-2	5.22 ± 1.07	3.93 ± 1.05*	< 4.950	83.08	68.97	0.778
Loi L	NR	6.70 ± 1.38	NA	NA	NA	NA

Note that for Meng N-1 and N-2, there may be cohort overlaps

NR = not reported

NA = not applicable

* = statistically significant difference reported between mean of malignant and benign lesions

### Determination of histopathological characteristics

Table 3 reports the correlation of APT signal intensity with various histopathological characteristics. Six studies evaluated the correlation between APT signal intensity and tumor grade with 3 studies reporting a positive correlation higher tumor grade and 3 studies reporting no correlation. One study reported a positive correlation between tumor stage and signal intensity. Seven studies evaluated the correlation between APT signal intensity and ER status with 1 study reporting a positive correlation with ER-negative status and 5 studies reporting no correlation. Seven studies evaluated the correlation between APT signal intensity and either PR or HER-2 status with all 7 studies reporting no correlation. Nine studies evaluated the correlation between APT signal intensity and Ki-67 expression and 3 reported a positive correlation with higher Ki-67 expression and 7 studies reported no correlation. One paper (Kamitami [27]) found that triple negative tumors had higher APT values than other subtypes. Specific APT values can be found in Supplemental Tables 2 and 3.

**Table 3:** APT correlation with histopathological characteristics

Paper	Tumor Grade/Stage	ER Status	PR Status	Her-2 Status	Ki67 Expression
Kamitani T	No	NR	NR	NR	Yes-positive*
Liu Z	Yes-positive (S)^a^*	No	No	No	Yes-positive*
Meng N-1	Yes-positive (G)*	No	No	No	No
Li Y	Yes-positive (G)*	No	No	No	No
Meng N-2	Yes-positive (G)*	No	No	No	No
Zhuang	No	No	No	No	No
Zhang N-1	NR	Yes-negative*	No	No	No
Yu	NR	No	No	No	No
Loi	NR	NR	NR	NR	No
Krikken-1	NR	No	No	No	NR

(S) staging; (G) grading; (NR) not reported

a = Tumor staging had the following sample sizes for T1/2/3/4: 14/26/12/2

* statistically significant difference reported (*p* < 0.05)

No correlation indicates no statistical difference in comparison

### Prediction of response to neoadjuvant chemotherapy

Four papers investigated changes in APT signal intensities before and after neoadjuvant chemotherapy (NAC). A case series of 3 patients found an increase in APT signal in a non-responder and partial responder and a decrease in signal in the 1 complete responder after one cycle of chemotherapy (Dula et al. [36]). Likewise, a study of 9 patients (10 lesions) showed that responders tend to have a lower APT signal after one cycle of chemotherapy compared to non-responders (Krikken E-1 [38]). Another study found that while APT signal intensity decreased in both responders and non-responders after 2 cycles of NAC, responders (*N* = 14) had a greater decrease in APT after NAC compared to non-responders (*N* = 36) (Zhang N-2 [34]). They also reported that pre-NAC APT signal intensity could predict response to NAC with an AUC of 0.690, while APT signal data pre- and post-NAC could predict response to NAC with an AUC of 0.879. Moreover, if tumor diameter data is included, the AUC increases to 0.903. Another study showed a significant decrease in APT signal at 0.9T but not 2.0T after the first two cycles of chemotherapy compared to baseline in the responders (*N* = 26), however, no difference in signal intensity in either strength was seen after the 4th cycle of chemotherapy compared to baseline [31]. No difference in signal intensity was noted between the pathological responders and non-responders at any point in time.

## Discussion

We review the use of APT to distinguish benign from malignant breast cancer lesions, characterize breast cancer lesions and predict breast cancer response to NAC. Our main findings are: (i) APT can distinguish benign from malignant breast cancer lesions with an AUC as high as 0.959, (ii) there is evidence that APT signal is positively correlated with a higher tumor grade/stage, although there are studies that report otherwise, (iii) there is no evidence to suggest a correlation between APT signal and ER/PR/Her-2 receptor status, (iv) there is weak evidence of a positive correlation between APT signal and higher Ki-67 expression, and (v) responders to NAC show a greater decrease in APT signal intensity after NAC compared to non-responders, although there are studies that report otherwise. Overall, these results suggest that APT has the potential to be used as a valuable imaging biomarker to differentiate benign from malignant breast cancer lesions and identify pathological responders to NAC.

### Malignant vs. Benign

Malignant tumors are recognized as having a higher APT signal intensity compared to benign tumors, which is consistent with prior studies of brain, gynecological, bladder, head and neck, and parotid gland tumors [23, 24, 40–43]. This heightened signal is thought to be due to a number of properties inherent to malignant tumors such as hypercellularity, fast cell turnover, and increased metabolic activity. Cells with faster turnover and increased metabolic activity have higher intracellular protein concentrations, amplifying the APT signal [13, 20, 44]. Likewise, the densely packed, hypercellular nature of malignant tumors further increases the local concentration of proteins, boosting signal intensity. Angiogenesis by malignant tumors may contribute to a higher signal too as the blood contains high concentrations of hemoglobin and albumin [13].

Four studies reported a higher APT signal intensity in malignant lesions compared to benign lesions, consistent with existing literature [26, 28, 30, 33]. However, the other 3 studies reported a higher APT signal in benign lesions [29, 32, 35]. One proposed mechanism for this finding is that physiologic breast secretions are rich in protein, and if these secretions are limited as in the case of an underlying malignancy, the number of proteins in the malignant lesions should decrease, as should the APT signal intensity [29, 35]. However, if this proposed mechanism were true, then high-grade malignant lesions would have a lower intensity than low grade lesions which is not observed in the aforementioned studies. Moreover, this hypothesis is not consistent with malignancies of other secretory organs, such as the parotid glands [23, 45]. Furthermore, malignant breast cancer cells are thought to secrete more proteins compared to benign cells in order to maintain a tumorigenic state [46]. Another possible reason for a higher APT signal in benign lesions could be due to variations in protein secretions between breast cancer subtypes. However, since all the studies have a heterogeneous sample of both benign and malignant breast cancer lesions, it is difficult to make conclusions regarding this hypothesis. Nonetheless, we implore future studies to report the APT signal intensity of each breast cancer subtype.

### APT correlation with histologic grade and Ki-67

Tumor histopathological characteristics are crucial in developing a treatment plan and determining prognosis for breast cancers but require an invasive biopsy. APT imaging may provide a non-invasive method of garnering histopathological information. In this review, all studies that investigated a correlation between APT signal intensity and Ki-67 found a positive correlation, though only two studies reached statistical significance. This is notable as Ki-67 has been a well-established marker of tumor proliferation and is utilized in therapeutic decision making [47]. Furthermore, prior studies have found that patients with low Ki-67 had significantly greater rates of survival and lower rates of metastases, though the full validity of Ki-67 as a robust prognostic marker is an ongoing area of research [2, 48]. It should be noted that different groups utilized different cutoffs for defining a high Ki-67. The two groups which found significant correlations utilized cutoffs of 20% and 30%, respectively, but the other groups did not find any correlations using a cut-off of 14%. At present, there is no well-established, clinically relevant cutoff for high and low Ki-67 statuses outside of levels less than 5% or greater than 30% [48]. Similarly, tumor grade plays an important prognostic role as higher grade tumors have worse prognosis than lower grade tumors [49]. All studies reported a higher signal intensity for higher graded/staged tumors with most of the studies reaching statistical significance.

Ultimately, tumor characteristics with worse prognosis, such as a high-Ki67, high histologic grade, and triple negative tumors appear to have higher APT signal intensities. Although APT imaging cannot provide definitive histopathological data, the presence of a high signal may be an early, non-invasive indication of a more severe malignancy which may aid clinicians in taking a more aggressive therapeutic approach. Future studies analyzing the ability of APT imaging to categorize lesions by tumor grade may be useful.

### APT signal of responders and non-responders to neoadjuvant chemotherapy

Early evaluation and prediction of the effectiveness of NAC is crucial in cancer treatment as it allows refinement of chemotherapy treatment prior to surgical intervention. One study in this review found a lower pretreatment ATP signal intensity to be a predictor of response to NAC, which is consistent with existing literature of rectal, cervical, and nasopharyngeal cancers [12, 50, 51]. The other studies found a difference between baseline APT signal intensity, which could be due to the smaller sample size in these studies. Most studies in this review suggest that responders to chemotherapy have a greater decrease in APT signal intensity than non-responders, though not all results reached statistical significance. This is consistent with a study of nasopharyngeal carcinoma, and other existing literature that suggests a decrease in APT is expected in responders approximately 6–7 days post-chemotherapy due to tumor necrosis [52, 53].

The one study that generated a predictive model to differentiate responders from non-responders found a high predictive value when using pre- and post-NAC APT signal intensity data [34]. The inclusion of tumor diameter data further increased the predictive value, suggesting that the use of APT imaging in conjunction with additional tumor characteristics may be a powerful method of differentiating responders from non-responders in the early stages of chemotherapy. The prediction using only pre-NAC data was not nearly as successful, despite other predictive models of rectal and cervical cancers using only pre-NAC APT signal intensity data showing promising results [12, 50]. The implementation of additional tumor characteristics, such as tumor diameter, to pre-NAC models in breast cancer has yet to be explored and could provide a reliable prediction even prior to the induction of chemotherapy.

### Comparison with other methods

Breast MRI has transitioned from relying mainly on contrast-enhanced imaging to a more multiparametric approach. Today, it commonly includes with and without fat-suppression, non-fat suppressed T1-weighted, and diffusion-weighted imaging as standard components. The multiparametric approach has allowed for increased diagnostic power of malignant lesions, especially in incidental lesion findings. However, the foundation of any breast MRI protocol remains the dynamic contrast-enhanced T1-weighted sequence [54, 55]. The T1-contrast enhanced sequence’s primary limitation remains the need for invasive intravenous contrast which may carry long term effects and is not suitable for all patients [54]. A novelty of the APT method lies in its ability to measure signals based on cytosolic protein changes which is distinct from other common contrast methods. Some studies have compared APT to other MRI methods in their ability to distinguish malignancy of lesions. Four groups reported that APT was at least as efficient in distinguishing malignancy when compared to other methods including DWI, DCE, intravoxel incoherent motion imaging (IVIM), and diffusion kurtosis imaging (DKI) [26, 30, 32, 33]. Of these groups, the AUC for APTw imaging ranged from 0.816 to 0.959. This was compared to AUCs of DCE ranging from 0.745 to 0.976 and DWI ranging from 0.878 to 0.897. There was one group whose analysis showed that APT was slightly inferior to DKI [35]. Interestingly, some groups also used APT as a supplement to other diagnostic methods and found that DCE combined with APT could yield a higher AUC for distinguishing malignant from benign lesions [26, 32, 35]. These results suggest that APT can be a supplemental MRI method in the multiparametric approach to amplify the diagnostic strength of traditional MRI contrast approaches or as an alternative to those with renal complications and pregnant patients.

### Limitations

There are several limitations in this study. First, variation in image processing and data analysis make interstudy comparison of APT signal intensity difficult. For instance, fat suppression is necessary given the high presence of adipose tissue in the breast. Without correction for the fat signal, there may be incorrect normalization of the APT signal or observation of pseudo-signals [56]. Studies used different techniques including short T1 inversion recovery, spectral pre-saturation with inversion recovery, water excitation, or simply placing the region of interest over the tumor to eliminate adipose signals. Furthermore, whereas some groups reported the APT signal intensity by conducting MTR asymmetry analysis, other studies conducted an additional Lorenztian fit analysis before reporting the signal intensity. Some studies did not directly report their method of calculating APT signal intensity.

APT signal intensity of breast cancer subtypes was not reported which could present potential confounders. For example, the secretory nature of the mucinous adenocarcinoma subtype may positively skew the overall malignant breast cancer APT signal intensity. Future studies should report the APT signal intensity of the breast cancer subtypes.

Lastly, unlike imaging of other organs such as the brain, breast cancer imaging is more susceptible to artifacts from respiratory and cardiac motion [57]. There is also much greater variability in tissue density and vascularity in the breast compared to the brain.

Of note, the literature regarding APT weighted detection of breast cancer lesions is still fairly limited, with fourteen papers reported in this review. The sample size of each study was also limited in size. When combined with the lack of acquisition and signal processing standardization between studies, this makes the current literature insufficient to make any confirmatory or statistically significant conclusions through meta analyses.

## Conclusion

APT signal can distinguish between malignant and benign breast cancer. More proliferative cancer with worse prognosis generally has higher APT signal intensities. There is some evidence that APT signal is associated with histologic grade, Ki-67 and pathological complete response to NAC. APT has the potential to complement with other imaging methods in the diagnosis, prognosis, and management of breast cancer. Additional studies and standardization of APT acquisition methods are needed.

## Electronic supplementary material

Below is the link to the electronic supplementary material.

Supplementary Material 1

Supplementary Material 2

Supplementary Material 3

Supplementary Material 4
