# Supplementary material for: Detection of breast cancer lesions using APT weighted MRI: a systematic review
Source: J Transl Med. 2025 Jan 31;23:141. doi: 10.1186/s12967-025-06153-7 (PMC11786454; doi:10.1186/s12967-025-06153-7)
Supplement: Supplementary file 1 — Supplementary Material 1 [file 12967_2025_6153_MOESM1_ESM.docx]

**SUPPLEMENTAL TABLES**

**Supplemental Table 1. Size and Histopathological Data of Studies**

| Study | Size (cm, malignant) | Size (cm, benign) | ER+ | PR+ | HER2+ | Ki67 high (% cutoff) | Histologic Grade I/II/III |
| --- | --- | --- | --- | --- | --- | --- | --- |
| Zhuang L | 3.31 ± 1.25 | 2.19 ± 2.40 | 20 | 20 | 9 | 25 (14) | 1/12/11 |
| Kamitami T | NR | NA | NR | NR | 8 | 35 (30) | NR |
| Liu Z | 3.7 ± 2.0 | 2.4 ± 2.1 | 30 | 32 | 15 | 40 (20) | 0/25/22 |
| Meng N-1 | 2.56 ± 1.14 | 2.24 ± 0.89 | 38 | 24 | 30 | 48 (14) | 22/30 (low/high) |
| Zhang N-1 | 2.25 ± 0.45 | 2.26 ± 0.77 | 30 | 23 | 11 | 30 (14) | 0/10/31 |
| Zhang S | NR | NA | NR | NR | NR | NR | NR |
| Li Y | 1.29 ± 0.35 | 1.09 ± 2.09 | 32 | 30 | 14 | 24 (NR) | 12/25 (low/high) |
| Yu T | 3.25 ± 1.59 | 3.65 ± 2.48 | 31 | 22 | 35 | 13 (20) | NR |
| Zhang N-2 | NR | NA | NR | NR | NR | NR | 1/33/16 |
| Meng N-2 | 2.7 ± 1.2 | 2.1 ± 0.8 | 43 | 29 | 36 | 55 (14) | 26/34 (low/high) |
| Dula AN | NR | NA | 1 | NR | NR | NR | NR |
| Loi et al. | NR | NA | NR | NR | NR | NR | NR |
| Krikken E-1 | 3.47 ± 2.73 | NA | 8 | 5 | 1 | NR | NR |
| Krikken E-2 | NR | NA | 10 | 10 | 6 | NR | NR |

Note that for histologic grade, some groups reported grade by I/II/III and others only distinguished high/low

NR = not reported

NA = not applicable, study did not measure benign lesion

**Supplemental Table 2. APT values for tumor grade, ER, PR, HER2, Ki67 expression**

| Paper | Tumor Grade | | ER Status | | PR Status | | HER2 Status | | Ki67 Expression | |
| --- | --- | --- | --- | --- | --- | --- | --- | --- | --- | --- |
|  | High | Low | + | - | + | - | + | - | High | Low |
| Kamitani T | 2.08±0.76 | 1.71±0.84 | NR | NR | NR | NR | NR | NR | 2.25±0.70^a^* | 1.60±0.79^a^* |
| Liu Z | 1.54±0.63^a,e^* | 0.81±0.64^a,e^* | 1.08±0.97 | 1.61±1.11 | 1.08±0.97 | 1.61±1.11 | 1.29±0.97 | 1.30±1.10 | 1.50±0.98^a,d^* | 0.73±1.07^a,d^* |
| Meng N-1 | 4.88±1.19^a^* | 4.13±0.73^a^* | 4.15±1.09 | 4.11±1.63 | 4.19±1.19 | 4.09±1.44 | 4.03±1.23 | 4.22±1.44 | 4.17±1.51 | 4.04±0.78 |
| Li Y | 2.18±0.76* | 1.30±0.69* | 1.61±0.83 | 1.60±0.85 | 1.67±0.81 | 1.32±0.86 | 1.40±0.90 | 1.73±0.82 | 1.73±0.85 | 1.37±0.74 |
| Meng N-2 | 4.28±1.23^a^* | 3.43±0.99^a^* | 3.95±1.14 | 4.24±1.11 | 4.38±0.96 | 3.92±1.23 | 4.13±1.19 | 4.03±1.08 | 4.18±1.07 | 3.77±1.25 |
| Krikken E-1^b^ | NR | NR | 0.0304 | 0.0317 | 0.0278 | 0.035 | 0.0494 | 0.0286 | NR | NR |
| Dula AN^c^ | NR | NR | 2.92 | 4.09 | NR | NR | NR | NR | NR | NR |

a: Additional Sens/Spec/AUC data is present in the paper

b: Mean was calculated on our own, paper utilized a nonstandard reporting metric

c: Only 3 patients had relevant data, mean was calculated on our own

d: Results only indicated significance for high vs low Ki67 in IBC-NST samples

e: Results reflect tumor staging by TNM system Stage 1 vs Stage 3

* Statistically significant difference

NR = not reported

**Supplemental Table 3. Additional Sensitivity, Specificity, and AUC in Histopathologic Data**

| Study | Characteristic | Cutoff | Sensitivity (%) | Specificity (%) | AUC |
| --- | --- | --- | --- | --- | --- |
| Liu Z | Ki67 high/low | NR | 67.5 | 72.6 | 0.696 |
|  | Stage 3/2 IBC-NST | NR | 90.9 | 52.0 | 0.702 |
| Kamitami T | Ki67 high/low | > 1.69 | 77.4 | 60 | 0.682 |
| Meng N-1 | Hist grade high/low | > 4.820 | 82.35 | 69.23 | 0.735 |
| Meng N-2 | Hist grade high/low | > 3.66 | 70 | 68.18 | 0.717 |

**Supplemental Table 4. APT values for prediction of response to NAC**

| Study | (+) pre-NAC mean | (+) post-NAC mean | (+) diff | (-) pre-NAC mean | (-) post-NAC mean | (-) diff |
| --- | --- | --- | --- | --- | --- | --- |
| Zhang N-2* | 2.66 | 1.23 | -1.44 | 3.13 | 2.51 | -0.58 |
| Krikken E-1** | 0.031 | 0.027 | -0.0049 | 0.025 | 0.030 | 0.0046 |

* Study showed significant difference between (+) diff and (-) diff

** Study found significant difference between (+) pre-NAC mean and (+) post-NAC
