# Supplementary material for: Detection of breast cancer lesions using APT weighted MRI: a systematic review
Source: J Transl Med. 2025 Jan 31;23:141. doi: 10.1186/s12967-025-06153-7 (PMC11786454; doi:10.1186/s12967-025-06153-7)
Supplement: Supplementary file 3 — Supplementary Material 3 [file 12967_2025_6153_MOESM3_ESM.docx]

**Manuscript ID: JTRM-D-24-13042**

**Manuscript Title: Detection of Breast Cancer Lesions Using APT Weighted MRI: A Systematic Review**

Author’s response to reviewer’s comments:

We sincerely appreciate the constructive feedback of the reviewers. Below, we provide a detailed, point-by-point response to each comment and question raised by the reviewers.

Point-by-point responses:

**Reviewer #1:**

The topic of the article is fascinating, yet the work is poorly organized. A considerable change to the structure is necessary. Please see my feedback below, which will assist in the improvement of the work.

(i) This review focuses on utilizing ATP-MRI to identify breast cancer. There should be a section dedicated to ATP-MRI that explains in depth how it works in cancer diagnosis, why it is significant, how it differs from CEST, and so on.

Thank you for the suggestion regarding a deeper introduction on APT theory. We have made significant additions to the introduction explaining in-depth how APT MRI functions. These additions supplement the current statements on its applications in detecting cancer and bridge the APT theory to clinical tumor detection. We also believe this novel contrast method is significant as its measured signal is different from the signal measured by more traditional contrast approaches (T2WI, DWI). This approach also does not require the use of nephrotoxic contrast agents, which may increase its use for at-risk patients. Both reasons have been added to the revised introduction. Finally, APT is noted to be a subset of CEST, which we have further emphasized in the revision.

**Highlighted** text revisions (Introduction):

Common breast MRI methods include Dynamic Contrast-Enhanced MRI (DCE-MRI), Diffusion-Weighted Imaging (DWI), T2-Weighted Imaging (T2WI), and less commonly used methods such as MR elastography and MR spectroscopy. **However, contrast agents are frequently required in the prior approaches which may present renal complications and contrast deposition in tissue [7,8].**

Chemical exchange saturation transfer (CEST) has been introduced as a novel contrast method which measures the exchange of protons between amide, amine, hydroxyl, and water groups [9]. **Of note, it does not require external contrast agents, which may expand its usefulness among patients with renal complications.** **While there are various approaches to CEST that measure different functional groups, a**~~A~~ specific subset of CEST measures the amide proton transfer (APT) at 3.5ppm away from the resonance frequency of water and has been widely investigated in various cancers [10-12]. **Amide proton transfer weighted imaging is sensitive to endogenous mobile proteins and peptides in tissue as opposed to the hydrogen atoms in free water which is measured in many other MR approaches. The principles and theories of CEST and APT weighted imaging have been reviewed in numerous articles [13-18]. In brief, the APT signal is observed as a decrease in the water signal caused by the exchange of water protons with tagged amide protons in mobile proteins. This provides indirect information about specific proteins through the water signal typically analyzed in imaging. The tagging is achieved by applying selective radiofrequency (RF) waves at the specific frequency of amide protons, approximately +3.5 ppm downfield of the water signal. The data is analyzed using a Z-spectrum, which shows how the water signal changes with and without RF saturation. However, effects such as direct water saturation (DS) and magnetization transfer contrast (MTC) can interfere with the measurements. These effects are separated using asymmetry analysis, which calculates the difference in the Z-spectrum at +3.5 ppm (amide protons) and -3.5 ppm (other effects), reported as MTRasym. A schematic of APT-weighted imaging is provided in Figure 1.**

**Since the 2000s, researchers have investigated the potential for APT imaging in cancer diagnosis.** In preclinical tumor models, increases in cytosolic protein concentrations have been found and correlated to increases in APT signal intensity [16,17]. A further study has found that approximately 66% of an APT signal change in tumors can be attributed to protein concentration differences **as malignant tumors typically have increased free protein due to angiogenesis and hypercellularity [18].** **This theory can be applied clinically as** ~~In clinical studies,~~ APT has been shown to be able to distinguish malignant from benign lesions, predict certain histologic grades, and correlate positively with response to neoadjuvant chemotherapy (NAC) response.

(ii) Why is the author solely concentrating on breast cancer? Is it used to treat other cancers? How does it work for secondary tumours?

We appreciate the reviewer’s comment. This special issue is on breast cancer. APT weighted imaging has been studied most extensively in brain cancers: its use has already been reviewed in brain tumors.

**Highlighted** text revisions (Introduction):

**APT weighted imaging applications have predominantly been investigated in brain tumors [19]. It has also been investigated in other cancers including salivary gland cancers, head and neck cancers, and even secondary metastases [20-22].** Its use in clinical practice is steadily increasing~~, as evidenced by its extensive study in brain tumors~~ which has led to the development of consensus recommendations for standardization across different centers and MRI vendors **for the study of brain tumors [19].**

In recent years, a few studies have investigated the use of APT MRI in breast cancer. Like studies in other cancers, they have also centered around distinguishing malignant from benign lesions, evaluating tumor characteristics, and predicting NAC response.Comprehensive review consolidating the body of research on APT MRI application to breast cancer is lacking. **We thus conducted this systematic review** ~~The goal of this systematic review is~~ to assess the diagnostic and prognostic use of APT in breast cancer and its future clinical applications.

(iii) The author identified certain drawbacks in the manuscript but did not compare them to other available methodologies. Furthermore, they do not give us the future prospects or commercial usefulness of this method.

Thank you for the insightful suggestion. We have made numerous additions to the manuscript to address other available methodologies. As detailed in our response to comment 1, we have included brief differences in signal source and need for contrast between APT and current MRI methods. In the results (**Table 1**), we have included notes on if each study compared efficacy of APT to other methodologies. Finally, we have furthered our discussion regarding the differences and limitations of other MRI methods in breast cancer detection and highlight where APT may fill these gaps. We believe that in the future, APT may prove a useful supplemental MRI method to amplify diagnostic accuracy and also be useful as a contrast method for those unable to tolerate traditional contrast agents which may be nephrotoxic. We believe these will be the future prospects and usefulness of this method, which we have also included in the revised text.

**Highlighted** text revisions (Discussion):

**Breast MRI has transitioned from relying mainly on contrast-enhanced imaging to a more multiparametric approach. Today, it commonly includes with and without fat-suppression, non-fat suppressed T1-weighted, and diffusion-weighted imaging as standard components. The multiparametric approach has allowed for increased diagnostic power of malignant lesions, especially in incidental lesion findings. However, the foundation of any breast MRI protocol remains the dynamic contrast-enhanced T1-weighted sequence [54, 55]. The T1-contrast enhanced sequence’s primary limitation remains the need for invasive intravenous contrast which may carry long term effects and is not suitable for all patients [54]. A novelty of the APT method lies in its ability to measure signals based on cytosolic protein changes which is distinct from other common contrast methods.** Some studies have ~~also~~ compared APT to other MRI methods in their ability to distinguish malignancy of lesions. Four groups reported that APT was at least as efficient in distinguishing malignancy when compared to other methods including DWI, DCE, **intravoxel incoherent motion imaging (IVIM), and diffusion kurtosis imaging (DKI) [26, 30, 32, 33].** **Of these groups, the AUC for APTw imaging ranged from 0.816 to 0.959. This was compared to AUCs of DCE ranging from 0.745 to 0.976 and DWI ranging from 0.878 to 0.897.** There was one group whose analysis showed that APT was slightly inferior to DKI [35]. Interestingly, some groups also used APT as a supplement to other diagnostic methods and found that DCE combined with APT could yield a higher AUC **for distinguishing malignant from benign lesions [26, 32, 35].** These results suggest that APT can be a supplemental MRI method **in the multiparametric approach** to amplify the diagnostic strength **of traditional MRI contrast approaches or as an alternative to those with renal complications and pregnant patients.**

(iv) This publication does not include a diagram. A schematic presentation helps the reader better understadent the material. Please include schematic presentations in your article.

We appreciate the suggestion for a schematic. We have added a figure outlining the theory of APT weighted imaging to further enhance understanding of the material.

**Highlighted** text revisions (Introduction):

**Figure 1: APT weighted imaging principle. A radiofrequency is applied at the specific frequency of amide protons 3.5ppm downfield the water signal. The exchange of amide protons with free water protons causes a change in the water signal. Asymmetry analysis around ± 3.5ppm is analyzed as the APT weighted signal and reported as MTRasym.**

**Reviewer #2:**

In the manuscript, Dr. Lee and coauthors conducted a systemic review of a novel MRI technique called APT that has shown promising ability to study cancers. This paper has conducted review about the application of APT into the diagnosis and prognosis of breast cancer, which has the ability to distinguish between benign and malignant tumor. Overall, this manuscript is well-written with good quality in language, scientific rationales and rigor, methods and results presented. I have several minor concerns are as follow:

1. In the abstract, I would like the authors to explicitly mention the prediction ability of APT to NAC, because this is an important finding and could be important to promote APT as a supplemental MRI method to the diagnosis of breast cancer.

Thank you for the comments. We agree that the prediction ability of APT to NAC is of particular interest as it could be used as a supplemental method in breast cancer management. We have modified the statement in the abstract to further clarify its potential as a supplemental method based on NAC results.

**Highlighted** text revisions (Abstract):

**There was a** greater decrease in APT signal intensity after neoadjuvant chemotherapy **(NAC)** in responders compared to non-responders, suggesting that APT MRI may serve as a valuable **supplemental** tool in the early identification of chemotherapy response.

2. It is also important to compare APT to other standard MRI approaches, such as T2WI and DWI the authors mentioned. I would like to see more introduction, results and discussion about them (currently I only found them in the discussion part)

We agree with the reviewer’s comments and have made numerous additions addressing other standard MRI approaches. In the introduction, we have added statements regarding the different contrast sources between APT and other standard approaches and highlighted how APT does not require the use of external contrast agents. Regarding the results section, we have included notes in **Table 1** on if each study compared APT to other standard MRI approaches and if they found it to be more/less/equally efficacious. In the discussion, we have furthered our discussion on the limitations of standard MRI contrast approaches. We believe APT can be a valuable supplemental MRI method to amplify diagnostic strength.

**Highlighted** text revisions (Discussion):

**Breast MRI has transitioned from relying mainly on contrast-enhanced imaging to a more multiparametric approach. Today, it commonly includes with and without fat-suppression, non-fat suppressed T1-weighted, and diffusion-weighted imaging as standard components. The multiparametric approach has allowed for increased diagnostic power of malignant lesions, especially in incidental lesion findings. However, the foundation of any breast MRI protocol remains the dynamic contrast-enhanced T1-weighted sequence [54, 55]. The T1-contrast enhanced sequence’s primary limitation remains the need for invasive intravenous contrast which may carry long term effects and is not suitable for all patients [54]. A novelty of the APT method lies in its ability to measure signals based on cytosolic protein changes which is distinct from other common contrast methods.** Some studies have ~~also~~ compared APT to other MRI methods in their ability to distinguish malignancy of lesions. Four groups reported that APT was at least as efficient in distinguishing malignancy when compared to other methods including DWI, DCE, **intravoxel incoherent motion imaging (IVIM), and diffusion kurtosis imaging (DKI) [26, 30, 32, 33].** **Of these groups, the AUC for APTw imaging ranged from 0.816 to 0.959. This was compared to AUCs of DCE ranging from 0.745 to 0.976 and DWI ranging from 0.878 to 0.897.** There was one group whose analysis showed that APT was slightly inferior to DKI [35]. Interestingly, some groups also used APT as a supplement to other diagnostic methods and found that DCE combined with APT could yield a higher AUC **for distinguishing malignant from benign lesions [26, 32, 35].** These results suggest that APT can be a supplemental MRI method **in the multiparametric approach** to amplify the diagnostic strength **of traditional MRI contrast approaches or as an alternative to those with renal complications and pregnant patients.**

3. In the introduction, I would like authors to elaborate rationale and background of APT, i.e. how does APT work.

We appreciate the suggestion and have made major additions to the introduction section. In brief, it provides a deeper explanation into how APT works and how it differs from existing contrast approaches. We have also included a visual schematic detailing the APT signal measurement.

**Highlighted** text revisions (Introduction):

Common breast MRI methods include Dynamic Contrast-Enhanced MRI (DCE-MRI), Diffusion-Weighted Imaging (DWI), T2-Weighted Imaging (T2WI), and less commonly used methods such as MR elastography and MR spectroscopy. **However, contrast agents are frequently required in the prior approaches which may present renal complications and contrast deposition in tissue [7,8].**

Chemical exchange saturation transfer (CEST) has been introduced as a novel contrast method which measures the exchange of protons between amide, amine, hydroxyl, and water groups [9]. **Of note, it does not require external contrast agents, which may expand its usefulness among patients with renal complications.** **While there are various approaches to CEST that measure different functional groups, a**~~A~~ specific subset of CEST measures the amide proton transfer (APT) at 3.5ppm away from the resonance frequency of water and has been widely investigated in various cancers [10-12]. **Amide proton transfer weighted imaging is sensitive to endogenous mobile proteins and peptides in tissue as opposed to the hydrogen atoms in free water which is measured in many other MR approaches. The principles and theories of CEST and APT weighted imaging have been reviewed in numerous articles [13-18]. In brief, the APT signal is observed as a decrease in the water signal caused by the exchange of water protons with tagged amide protons in mobile proteins. This provides indirect information about specific proteins through the water signal typically analyzed in imaging. The tagging is achieved by applying selective radiofrequency (RF) waves at the specific frequency of amide protons, approximately +3.5 ppm downfield of the water signal. The data is analyzed using a Z-spectrum, which shows how the water signal changes with and without RF saturation. However, effects such as direct water saturation (DS) and magnetization transfer contrast (MTC) can interfere with the measurements. These effects are separated using asymmetry analysis, which calculates the difference in the Z-spectrum at +3.5 ppm (amide protons) and -3.5 ppm (other effects), reported as MTRasym. A schematic of APT-weighted imaging is provided in Supplemental Figure 1.**

**Since the 2000s, researchers have investigated the potential for APT imaging in cancer diagnosis.** In preclinical tumor models, increases in cytosolic protein concentrations have been found and correlated to increases in APT signal intensity [16,17]. A further study has found that approximately 66% of an APT signal change in tumors can be attributed to protein concentration differences **as malignant tumors typically have increased free protein due to angiogenesis and hypercellularity [18].** **This theory can be applied clinically as** ~~In clinical studies,~~ APT has been shown to be able to distinguish malignant from benign lesions, predict certain histologic grades, and correlate positively with response to neoadjuvant chemotherapy (NAC) response.

**Figure 1: APT weighted imaging principle. A radiofrequency is applied at the specific frequency of amide protons 3.5ppm downfield the water signal. The exchange of amide protons with free water protons causes a change in the water signal. Asymmetry analysis around ± 3.5ppm is analyzed as the APT weighted signal and reported as MTRasym.**

4. In the Table 3 correlation analysis, it is not clear to me what does no correlation means. Does no correlation mean the correlation is very close to zero and not statistically significant. Does positive correlation mean that it is statistically significant? I would like authors to add footnotes to clearly state that.

Thank you for this suggestion. We have added asterisks to indicate statistical significance in reported correlations to the table and the appropriate footnote. One of the reported “Yes-positive” results by Loi et al. was amended to “No” as it was reported as a positive correlation, though statistical analysis did not show significance (p < 0.05). For consistency of the table, we decided to indicate “No” as it did not reach significance.

**Highlighted** text revisions (Results):

Table 3. APT Correlation With Histopathological Characteristics

| Paper | Tumor Grade/Stage | ER Status | PR Status | Her-2 Status | Ki67 Expression |
| --- | --- | --- | --- | --- | --- |
| Kamitani T | No | NR | NR | NR | Yes-positive***** |
| Liu Z | Yes-positive (S)^a^***** | No | No | No | Yes-positive***** |
| Meng N-1 | Yes-positive (G)***** | No | No | No | No |
| Li Y | Yes-positive (G)***** | No | No | No | No |
| Meng N-2 | Yes-positive (G)***** | No | No | No | No |
| Zhuang | No | No | No | No | No |
| Zhang N-1 | NR | Yes-negative***** | No | No | No |
| Yu | NR | No | No | No | No |
| Loi | NR | NR | NR | NR | **No** |
| Krikken-1 | NR | No | No | No | NR |

(S) = staging; (G) grading; **(NR) not reported**

a = Tumor staging had the following sample sizes for T1/2/3/4: 14/26/12/2

*** statistically significant difference reported (p <0.05)**

**No correlation indicates no statistical difference in comparison**

5. Citations are needed for the paragraphs of lines 165-171 and lines 174-179.

Thank you for your careful review. We have added the following citations for lines 165-171: (1)(2)(3) and the following citations for lines 174-179: (4)(5)

(1) Zhou J, Heo HY, Knutsson L, van Zijl PCM, Jiang S. APT-weighted MRI: Techniques, current neuro applications, and challenging issues. J Magn Reson Imaging. 2019 Aug;50(2):347-364. doi: 10.1002/jmri.26645
(2) Lee DH, Heo HY, Zhang K, Zhang Y, Jiang S, Zhao X, Zhou J. Quantitative assessment of the effects of water proton concentration and water T1 changes on amide proton transfer (APT) and nuclear overhauser enhancement (NOE) MRI: The origin of the APT imaging signal in brain tumor. Magn Reson Med. 2017 Feb;77(2):855-863. doi: 10.1002/mrm.26131
(3) Yan K, Fu Z, Yang C, Zhang K, Jiang S, Lee DH, Heo HY, Zhang Y, Cole RN, Van Eyk JE, Zhou J. Assessing Amide Proton Transfer (APT) MRI Contrast Origins in 9 L Gliosarcoma in the Rat Brain Using Proteomic Analysis. Mol Imaging Biol. 2015 Aug;17(4):479-87. doi: 10.1007/s11307-015-0828-6
(4) Meng N, Wang X, Sun J, Han D, Bai Y, Wei W, Wang Z, Jia F, Wang K, Wang M. A comparative study of the value of amide proton transfer-weighted imaging and diffusion kurtosis imaging in the diagnosis and evaluation of breast cancer. Eur Radiol. 2021 Mar;31(3):1707-1717. doi: 10.1007/s00330-020-07169-x
(5) Meng N, Wang XJ, Sun J, Huang L, Wang Z, Wang KY, Wang J, Han DM, Wang MY. Comparative Study of Amide Proton Transfer-Weighted Imaging and Intravoxel Incoherent Motion Imaging in Breast Cancer Diagnosis and Evaluation. J Magn Reson Imaging. 2020 Oct;52(4):1175-1186. doi: 10.1002/jmri.27190

**Highlighted** text revisions (Discussion):

165-171:

This heightened signal is thought to be due to a number of properties inherent to malignant tumors such as hypercellularity, fast cell turnover, and increased metabolic activity. Cells with faster turnover and increased metabolic activity have higher intracellular protein concentrations, amplifying the APT signal **[12, 35, 36]**. Likewise, the densely packed, hypercellular nature of malignant tumors further increases the local concentration of proteins, boosting signal intensity. ~~The lower extracellular pH of malignant tumors may also contribute to a greater APT signal by enhancing the exchange rate of amide protons.~~ **Angiogenesis by malignant tumors may contribute to a higher signal too as the blood contains high concentrations of hemoglobin and albumin [35].**

174-179:

One proposed mechanism for this finding is that physiologic breast secretions are rich in protein, and if these secretions are limited as in the case of an underlying malignancy, the number of proteins in the malignant lesions should decrease, as should the APT signal intensity **[18, 24]**.

6. One of the limitation of this systemic review is that the number of literatures is small (only around 15) and the sample size of each study is too small to make any confirmatory and statistically significant conclusions. I would like authors to mention this point in the limitation.

I am very impressed with the great quality of written and scientific insights behind this systemic review. It's interesting for me to see APT intensity could be a predictor to NAC with a high AUC, which indicates that APT could be an attractive technique in the future for the diagnosis and prognosis of breast cancer

We appreciate the reviewer’s positive feedback. We agree that the limited state of the literature and small sample sizes limit the ability to make statistically significant conclusions. We have now added an explicit acknowledgement of this particular limitation.

**Highlighted** text revisions (Discussion):

"**Of note, the literature regarding APT weighted detection of breast cancer lesions is still fairly limited, with fourteen papers reported in this review. The sample size of each study was also limited in size. When combined with the lack of acquisition and signal processing standardization between studies, this makes the current literature insufficient to make any confirmatory or statistically significant conclusions through meta analyses.**"
