# Supplementary material for: Detection of breast cancer lesions using APT weighted MRI: a systematic review
Source: J Transl Med. 2025 Jan 31;23:141. doi: 10.1186/s12967-025-06153-7 (PMC11786454; doi:10.1186/s12967-025-06153-7)
Supplement: Supplementary file 4 — Supplementary Material 4 [file 12967_2025_6153_MOESM4_ESM.docx]

To:

Dr. Mary Salvatore, MD, MBA

SI editors of Prevention and Early Diagnosis of Breast Cancer in Journal of Translational Medicine

Subject: Submission to the special issue on Prevention and Early Diagnosis of Breast Cancer

Title: Detection of Breast Cancer Lesions Using APT Weighted MRI: A Systematic Review

Dear Editors and Reviewers,

We thank you for the reviews of our manuscript. Please find the point-by-point responses and revised manuscripts with and without track changes.

Sincerely,

Tim Q. Duong, PhD

Email: tim.duong@einsteinmed.edu

Telephone: 718-920-5113

Address: Montefiore Medical Center, 111 East 210th Street, Bronx, NY 10467
